# *ESR1 *and *EGF *genetic variation in relation to breast cancer risk and survival

**DOI:** 10.1186/bcr1861

**Published:** 2008-02-14

**Authors:** Kristjana Einarsdóttir, Hatef Darabi, Yi Li, Yen Ling Low, Yu Qing Li, Carine Bonnard, Arvid Sjölander, Kamila Czene, Sara Wedrén, Edison T Liu, Per Hall, Keith Humphreys, Jianjun Liu

**Affiliations:** 1 Centre for Health Services Research, School of Population Health, University of Western Australia, 6009 Crawley, Perth, Western Australia; 2 Department of Medical Epidemiology and Biostatistics, Karolinska Institute, 171 77 Stockholm, Sweden; 3 Human Genetics, Genome Institute of Singapore, Singapore 138672; 4 Cancer Biology, Genome Institute of Singapore, Singapore 138672

## Abstract

**Introduction:**

Oestrogen exposure is a central factor in the development of breast cancer. Oestrogen receptor alpha (ESR1) is the main mediator of oestrogen effect in breast epithelia and has also been shown to be activated by epidermal growth factor (EGF). We sought to determine if common genetic variation in the *ESR1 *and *EGF *genes affects breast cancer risk, tumour characteristics or breast cancer survival.

**Methods:**

We genotyped 157 single nucleotide polymorphisms (SNPs) in *ESR1 *and 54 SNPs in *EGF *in 92 Swedish controls and selected haplotype tagging SNPs (tagSNPs) that could predict both single SNP and haplotype variation in the genes with an *R*^2 ^of at least 0.8. The tagSNPs were genotyped in 1,590 breast cancer cases and 1,518 controls, and their association with breast cancer risk, tumour characteristics and survival were assessed using unconditional logistic regression models, Cox proportional hazard models and haplotype analysis.

**Results:**

The single tagSNP analysis did not reveal association evidence for breast cancer risk, tumour characteristics, or survival. A multi-locus analysis of five adjacent tagSNPs suggested a region in *ESR1 *(between rs3003925 and rs2144025) for association with breast cancer risk (p = 0.001), but the result did not withstand adjustment for multiple comparisons (p = 0.086). A similar region was also implicated by haplotype analyses, but its significance needs to be verified by follow-up analysis.

**Conclusion:**

Our results do not support a strong association between common variants in the *ESR1 *and *EGF *genes and breast cancer risk, tumour characteristics or survival.

## Introduction

Breast cancer is the most common cancer in women overall worldwide. Oestrogen exposure is a central factor in the development and progression of this cancer [1-3] and its effects on the breast epithelium is primarily mediated by oestrogen receptor alpha (ESR1) [4]. In addition to being activated by oestrogen, the ESR1 protein can be activated by growth factors such as epidermal growth factor (EGF) [3], which acts as a potent mitogen for epithelial cells, including mammary epithelia [5]. Variation in the *ESR1 *(MIM 133430) and *EGF *(MIM 131530) genes affecting the function or expression of their respective proteins could thus potentially affect the risk of developing breast cancer, characteristics of the tumour or the risk of dying from the disease.

With regard to breast cancer risk or survival, a number of single nucleotide polymorphisms (SNPs) have been studied in the *ESR1 *gene, yet none have previously been investigated in the *EGF *gene. As far as we are aware, no attempt to capture the common genetic variation in the *ESR1 *gene in its entirety has yet been published. One group, who genotyped 17 SNPs in the *ESR1 *gene, found a decreased risk of breast cancer for carriers of three common haplotypes in the gene and an increased risk for carriers of one common haplotype [6]. We genotyped 157 SNPs in *ESR1 *and 54 SNPs in *EGF *using a population-based case-control study, which included 1,590 breast cancer cases and 1,518 controls. We selected haplotype-tagging SNPs (tagSNPs) spanning the *ESR1 *and *EGF *genomic regions and assessed their association with breast cancer risk, the Nottingham Prognostic Index (NPI) and breast cancer survival.

## Patients and methods

### Parent breast cancer study

The study base included all Swedish-born women between 50 and 74 years of age and resident in Sweden between October 1993 and March 1995. During this period, all breast cancer cases were identified at diagnosis through the six regional cancer registries in Sweden. Controls were randomly selected from the Swedish Registry of Total Population to match the cases in 5-year age strata. Of the eligible cases and controls, 3,345 (84%) breast cancer cases and 3,454 (82%) controls participated in this initial questionnaire-based study.

### Present breast cancer study

From the parent study, we randomly selected 1,500 breast cancer cases and 1,500 age- and frequency-matched controls among the postmenopausal participants without any previous malignancy (except carcinoma *in situ *of the cervix or non-melanoma skin cancer). With the intention of increasing statistical power in subgroup analyses, we further selected all remaining breast cancer cases and controls that had used menopausal hormones (oestrogen alone or any combination of oestrogen and progestin) for at least 4 years. We also included all remaining participants with self-reported diabetes mellitus. In total, we selected 1,801 breast cancer cases and 2,057 controls.

Following informed consent, participants donated whole blood. For deceased cases and those cases that declined to donate blood but consented to our use of tissue, we collected archived paraffin-embedded, non-cancerous tissue samples. We acquired 70% of the requested tissue samples; the main reason for non-participation was unwillingness or lack of time at the respective pathology department to provide the tissue blocks. In total, we obtained blood samples and archived tissue samples for 1,321 and 275 breast cancer patients, respectively, and blood samples for 1,524 controls. Population-based participation rates (taking into account the proportion that did not participate in the parent questionnaire study) were 75% and 61% for the cases and controls, respectively.

We extracted DNA from 4 ml of whole blood using the QIAamp DNA Blood Maxi Kit (Qiagen, Hilden, Germany) according to the manufacturer's instructions. From non-malignant paraffin-embedded tissues, DNA was extracted using a standard phenol/chloroform/isoamyl alcohol protocol [7]. We successfully isolated DNA from 1,318 (blood) and 272 (tissue) breast cancer patients and 1,518 controls. We randomly selected 92 out of the 1,518 controls to be used for linkage disequilibrium characterisation and haplotype reconstruction of the *ESR1 *and *EGF *genes.

This study was approved by the Institutional Review Boards in Sweden and at the National University of Singapore.

### SNP markers and genotyping

We selected SNPs in the *ESR1 *and *EGF *genes and their 20 kb flanking sequences from dbSNP (build 124, [8]) and Celera databases, aiming for an initial marker density of at least one SNP per 5 kb. The *Pvu*II (rs2234693), *Xba*I (rs9340799), codon 243 (rs4986934) and codon 325 (rs1801132) variants were selected from the literature and added to our SNP selection. SNPs were genotyped using the Sequenom primer extension-based assay (San Diego, CA, USA) and the BeadArray system from Illumina (San Diego, CA, USA) following the manufacturers' instructions. All genotyping plates included positive and negative controls, DNA samples were randomly assigned to the plates, and all genotyping results were generated and checked by laboratory staff unaware of case-control status. Only SNPs where more than 85% of the samples gave a genotype call were analysed further. As quality control, we genotyped 200 randomly selected SNPs in the 92 control samples using both the Sequenom system and the BeadArray system. The genotype concordance was > 99.5%, suggesting high genotyping accuracy.

### Linkage disequilibrium characterisation and tagSNP selection

We genotyped a dense set of SNPs in the *ESR1 *and *EGF *genes in the 92 controls (Supplementary Tables 1 and 2 in Additional File 1, respectively). We identified regions of linkage disequilibrium (LD) and selected tagSNPs. We produced LD plots of the *D' *and *R*^2 ^values for *ESR1 *and *EGF *(Supplementary Figures 1 and 2 in Additional File 1, respectively) using the *LDheatmap *function in the statistical software R [9]. We reconstructed haplotypes using the partition ligation expectation maximisation (PLEM) algorithm [10] implemented in the *tagSNPs *program [11] and selected tagSNPs based on the *R*^2 ^coefficient, described previously (equation (1) in [12]. In our case this is the squared correlation between the true number of haplotypes (defined across all SNPs typed in the 92 controls) and the number of copies of haplotypes predicted as being carried, based on the tagSNPs. The *R*^2 ^coefficient in [12] can also be used for measuring association between the genotypes of all SNPs typed in the 92 controls and the genotypes predicted on the basis of knowing the tagSNPs only. We chose tagSNPs so that common SNP genotypes (minor allele frequency ≥ 0.03) and common haplotypes (frequency ≥ 0.03) were predicted with *R*^2 ^≥ 0.8 [13]. The well studied *Pvu*II (rs2234693), *Xba*I (rs9340799), codon 243 (rs4986934) and codon 325 (rs1801132) variants were included as tagSNPs. In order to evaluate our tagSNPs' performance in capturing unobserved SNPs within the genes and to assess whether we needed a denser set of markers, we performed a SNP-dropping analysis [12,14]. In brief, each of the genotyped SNPs was dropped in turn and tagSNPs were selected from the remaining SNPs so that their haplotypes predicted the remaining SNPs with an *R*^2 ^value of 0.85. We then estimated how well the tagSNP haplotypes of the remaining SNPs predicted the dropped SNP, an evaluation that can provide an unbiased and accurate estimate of tagSNP performance [12,14].

There were 19 SNPs upstream of the first tagSNP (TAG1) in *ESR1 *(Supplementary Table 1 in Additional File 1). Of the 19, 12 were either not polymorphic or had a minor allele frequency (MAF) of less than 3%. The remaining seven SNPs in this area were included in our LD identification and tagSNP selection analysis. Hence, all polymorphic SNPs with a MAF ≥ 3% far 5' upstream of *ESR1 *were captured by our tagSNPs.

### Breast tumour characteristics and follow-up

We retrieved information on date and cause of death until 31 December 2003 from the Swedish Causes of Death Registry and on date of emigration from the Swedish National Population Registry. Follow-up time began at date of diagnosis and ended on 31 December 2003, or at date of death or emigration, whichever came first.

We collected information on tumour size, lymph node involvement, and grade (tumour differentiation) from medical records and calculated the Nottingham Prognostic Index (NPI) using the following formula:

NPI = 0.2 × size [in cm] + 1 × nodal stage [1, 2, or 3] + 1 × grade [1, 2, or 3] [15]

Nodal stage was defined as 1 if there were no lymph node metastases, 2 for a total of 1–3 metastatic nodes, and 3 for more than 3 metastatic nodes. A tumour of high differentiation was assigned grade 1, a tumour of intermediate differentiation grade 2, and a low differentiated tumour was assigned grade 3. We categorised the NPI into two groups: ≤ 4 or > 4. Four is the mean NPI value of the present study. It has also been shown that breast cancer survival decreases rapidly for NPI above 4 [15].

### Statistical analyses

We applied unconditional logistic regression models for assessing the association between *ESR1 *and *EGF *tagSNPs and risk of breast cancer (case-control analysis) or the NPI (case only analysis). Adjusting for age (in 5-year age groups) did not affect our results. We estimated the hazard ratio of death due to breast cancer in relation to the genes' tagSNP using Cox proportional hazards models. The tagSNPs were included as covariates in the models either one at a time or in groups of five (codominant main effects only). The latter method was used for detection of association with haplotypes. Although it does not require resolution of gametic phase, tests based on such models can be powerful within regions of strong LD [16]. Likelihood ratio tests were used to generate p values for comparing models with or without covariates. We made adjustments to our test results to account for multiplicity. We did so for each outcome (risk, NPI, and survival) separately. We used a permutation-based approach that controls the family-wise error rate (probability of rejecting one or more true null hypotheses of no association). This is based on the permutation step-down procedure of Westfall and Young [17] and takes into account the dependence structure of the polymorphisms/hypotheses. We also assessed association between groups of haplotypes and breast cancer risk using three approaches (each of which resolve gametic phase). We used the logistic regression expected haplotype dosage approach of [11], combining rare haplotypes. Since there is no biological reason to cluster haplotypes on the basis of their frequency, we also employed a Bayesian association mapping approach [18] that clusters haplotypes according to their allelic similarity, and a sliding-window approach described by Li *et al*. [19].

To estimate power in the risk component of the study, we used a method described by Chapman *et al*. [20], which assumes co-dominant effects at an unobserved locus. To calculate power for log-additive effects in the survival component of the study, we used the Quanto program [21] in a similar manner as Manolio *et al*. [22]. Analyses were performed using the statistical software R or the SAS system (v. 9.1, SAS Institute Inc., Cary, NC, USA). Because lifestyle and reproductive breast cancer risk factors are unlikely to cause genetic variation in the genes, we thus did not adjust for them in the analyses.

## Results

### Characteristics of participants

Table 1 shows selected characteristics of the cases and controls included in the parent questionnaire-based study and the current genetic study. Long-term users of menopausal hormone therapy and women with self-reported diabetes mellitus were oversampled in the current study. Most other characteristics were statistically significantly different between cases and controls and reflected established associations.

**Table 1:** Selected characteristics of the cases and controls participating in the present and parent breast cancer study

	Present		Parent	
	
Characteristic	No. of cases/controls	Cases/controls	No. of cases/controls	Cases/controls
		Mean:		Mean:
Age (years)	1,590/1,518	63.4/63.1	2,817/3,111	63.4/64.3
Age at menopause (years)	1,580/1,505	50.4/50.0	2,802/3,093	50.4/50.0
Recent BMI (kg/m^2^)^a^	1,581/1,497	25.8/25.5	2,802/3,065	25.8/25.5
Age at first birth (years)	1,352/1,370	25.4/24.7	2,373/2,753	25.3/24.6
Parity	1,590/1,518	1.8/2.2	2,817/3,110	1.8/2.1
Duration of menopausal hormone use (years)		Percentage:		Percentage:
0	1,058/1,086	67.2/72.7	1,978/2,467	71.4/80.8
< 4	206/190	13.1/12.7	405/330	14.6/10.8
≥ 4	311^b^/217^b^	19.8^b^/14.5^b^	389/256	14.0/8.4
Self-reported diabetes mellitus (yes/no)	1,588/1,402	9.0^b^/7.8^b^	2,810/2,652	6/6.1
Family history (yes/no)^c^	1,551/1,380	16.1/9.3	2,744/2,607	16.0/9.2
High NPI (≤ 4/> 4)	975/-	55.7/-		-/-

BMI, body mass index; NPI, Nottingham Prognostic Index. ^a^ At 1 year prior to diagnosis. ^b^ Long-term users of menopausal hormones and women with diabetes mellitus were oversampled. ^c^ Family history is defined as having at least one first degree relative with breast cancer.

More case-related information has been provided in our previous work [23]. The breast cancer cases that participated in our study via tissue sample donation were on average 1.5 years older (p = 0.0003) than the cases that donated blood. The former group was also more likely to have been diagnosed with TNM (tumour, nodes, metastasis) stage 2 or more advanced cancers (p < 0.0001). Since no significant differences in genotype frequencies within TNM stage 1, TNM stage 2 and TNM stages 3 and 4 were evident between the two groups of cases, this difference is unlikely to be a cause for concern.

### Genotyping, LD pattern and coverage

The genotyping results and SNP coverage in the *ESR1 *and *EGF *genes are summarised in Table 2. A dense set of SNPs in the *ESR1 *and *EGF *genes were genotyped in 92 randomly selected controls (Supplementary Table 1 (*ESR1*) and Supplementary Table 2 (*EGF*) in Additional File 1), and only the SNPs that were in Hardy-Weinberg equilibrium (p > 0.01) and that were at least 3% in minor allele frequency among the 92 controls were included in LD analysis and tagSNP selection (Table 2). LD plots created from the SNPs included in our study are shown in Supplementary Figures 1 (*ESR1*) and 2 (*EGF*) in Additional File 1. Using the SNP dropping method [14], we found that the tagSNPs selected from the included SNPs could efficiently capture non-genotyped SNPs in the genes (Table 2).

**Table 2:** Summary statistics on genotyping results and SNP coverage in *ESR1 *and *EGF *for 92 Swedish controls

Summary statistics	*ESR1*	*EGF*
Number of successfully genotyped SNPs	228^a^	104^b^
Number of polymorphic SNPs	184	66
Number of common SNPs^c^	165	55
Number of SNPs deviating from HWE^d^	8	1
		
Number of SNPs included in study	157	54
		
Gene size (kb)	295.7	99.4
Sequence coverage of included SNPs (kb)	335.1	145.5
Mean spacing between included SNPs (kb)	2.1	2.7
Median spacing between included SNPs (kb)	1.8	2.3
Number of tagSNPs selected	52	15
Average tagSNP prediction of common SNPs included in study (*R*^2^)^c^	0.998	0.987
		
Coverage evaluation^e^		
Average prediction of dropped SNPs (*R*^2^)	0.997	0.948
Percentage of *R*^2 ^values ≥ 0.7	100	96.3

HWE, Hardy-Weinberg equilibrium; SNP, single nucleotide polymorphism. ^a^ See Supplementary Table 1. ^b^ See Supplementary Table 2. ^c^ Common was defined as minor allele frequency ≥ 0.03. ^d^ p < 0.01. ^e^ SNP dropping method by Weale *et al*. [14].

### Association analyses

We selected 52 tagSNPs in *ESR1 *and 15 tagSNPs in *EGF *that could predict the included SNPs and their haplotypes with an *R*^2 ^of at least 0.8. The tagSNPs were genotyped in all cases and controls (Supplementary Table 3 in Additional File 1), but seven tagSNPs in *ESR1 *and one tagSNP in *EGF *could not be genotyped in the cases that participated via tissue sample donation.

### ESR1

**Figure 1:**
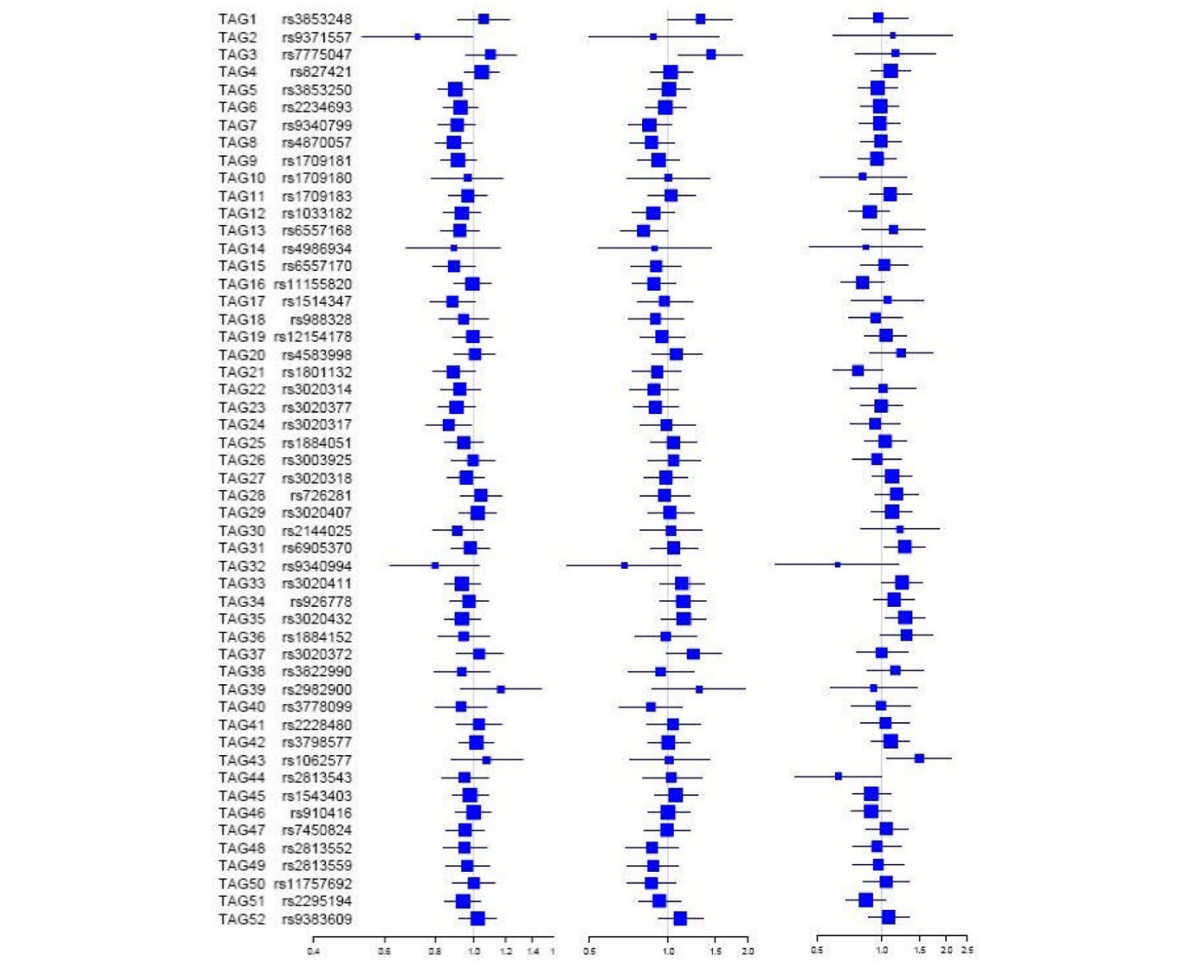
Association of 52 tagging single nucleotide polymorphisms (tagSNPs) in *ESR1 *with breast cancer risk, Nottingham Prognostic Index (NPI) and breast cancer survival. Left column: breast cancer risk. Middle column: NPI (case-only analysis). Right column: breast cancer survival. Squares and horizontal lines represent odds and hazard (survival analysis) ratios (change in risk with each addition of the rare allele) and their confidence intervals. Sizes of the squares reflect the minor allele frequencies. NPI was categorised into ≤ 4 or > 4.

For each outcome (breast cancer risk, NPI and breast cancer survival), we first tested the association of each tagSNP and then performed a haplotype analysis using a logistic regression sliding-window approach where five adjacent tagSNPs were analysed together (without resolution of gametic phase). The results are summarised in Figure 1 and Supplementary Table 4 in Additional File 1. Analysis of the 52 tagSNPs in *ESR1 *(including the *Pvu*II, *Xba*I, codon 243 and codon 325 SNPs) did not reveal any association with breast cancer risk, NPI or breast cancer survival whose statistical significance withstood multiple testing correction. The strongest signal of association with breast cancer risk was obtained by the window analysis including TAGs 26–30 (p = 0.001 and p = 0.086, before and after correction for multiple testing). Within the region, there were seven common haplotypes that accounted for 92% of the chromosomes. Including the expected dosages of common haplotypes and the rare haplotypes (combined into a single variable) as covariates in a logistic regression model with the most common haplotype as reference, gave a global p (likelihood ratio test) of 0.0493 in relation to breast cancer risk (Table 3).

**Table 3:** Association between haplotypes reconstructed from *ESR1 *TAGs 26–30 and breast cancer risk

		Haplotype proportions		
			
TAGs 26–30	Haplotypes	Cases (*n *= 1,579^a^)	Controls (*n *= 1,514^a^)	OR (95% CI)
Haplotype 1	ACAAC	0.57	0.56	1.00 (Reference)
Haplotype 2	GTGGT	0.09	0.10	0.91 (0.76–1.09)
Haplotype 3	ACAGC	0.06	0.06	1.05 (0.84–1.31)
Haplotype 4	ATGGT	0.06	0.06	0.89 (0.70–1.13)
Haplotype 5	ATGGC	0.06	0.05	1.05 (0.82–1.35)
Haplotype 6	GTGGC	0.05	0.03	1.46 (1.08–1.98)
Haplotype 7	GTAAC	0.03	0.04	0.72 (0.55–0.96)
	Rare^b^	0.08	0.08	0.94 (0.76–1.15)
Global p value^c^				0.0493

CI, confidence interval; SNP, single nucleotide polymorphism. ^a^ Information on at least one of the five tagSNPs. ^b^ Total of 18 rare haplotypes combined. Each haplotype has frequency below 3% among the controls. ^c^ Likelihood ratio test.

We also explored a sliding-window analysis of haplotypes using a variable window size. Three haplotypes within the region from tagSNP 18 to tagSNP 27 were implicated, showing frequency differences between cases and controls (Table 4). The significance of the frequency differences was, however, not clear, given the large number of haplotypes being searched in both fixed- and variable-sized sliding-window analyses. We also used a Bayesian haplotype clustering method [18], with a fixed window size of six tagSNPs. Interestingly, the posterior distribution for the position of a possible disease mutation coincided with the region suggested by both fixed- and variable-sized haplotype analyses (Supplementary Figure 3 in Additional File 1).

**Table 4:** Association of three haplotypes (TAGs 18–27) in *ESR1 *with breast cancer risk, as implicated by the variable-sized sliding-window analysis

tagSNPs	Haplotype	Frequency in cases	Frequency in controls	OR (95% CI)	p Value^a^
TAG18–21	ACAC	0.0798	0.0595	1.39 (1.13–1.69)	0.0014
TAG18–24	ACAGCGC	0.0456	0.0562	0.70 (0.56–0.88)	0.0019
TAG18–27	GCAGCGCGGT	0.0697	0.0813	0.85 (0.70–1.03)	0.0933

CI, confidence interval; SNP, single nucleotide polymorphism. ^a^ One vs the others Chi-squared test.

Also, an analysis within groups of diabetes mellitus, menopausal hormone use or family history furthermore did not reveal any significant evidence for any tagSNP to be associated with breast cancer risk (data not shown).

### EGF

None of the tagSNPs in *EGF *showed association with breast cancer risk, NPI, or breast cancer survival that withstood multiple testing correction (Supplementary Figure 4 in Additional File 1). This lack of association was supported by the haplotype analysis.

## Discussion

We had comprehensive SNP coverage of the entire *ESR1 *and *EGF *genes and were thus able to study if there were any common variants in the genes that showed an association with breast cancer risk, NPI or breast cancer survival. No association was found between common variants in *ESR1 *and NPI or breast cancer survival by single tagSNP analysis. A region between TAG26 (rs3003925) and TAG30 (rs2144025) in the *ESR1 *gene showed a signal for association with breast cancer risk in the multi-locus analysis of five adjacent tagSNPs, but the result did not withstand multiple testing correction. Interestingly, the suggestive evidence from further haplotype analyses converge to this region, but its significance needs to be determined by follow-up analysis. None of the genotyped SNPs within this region were located in exons (all were in the middle of intron 4–5) and are thus unlikely to affect ESR1 protein structure. It is still a possibility however that the SNPs themselves, or one or more SNPs in LD with any of the SNPs, may effect the regulation of ESR1 protein expression. In fact, it has been shown that ESR1 protein overexpression is common in breast cancer [24]. Common variants within the *EGF *gene did not appear to affect the risk of developing breast cancer, developing a tumour with high NPI, or dying from the disease.

Our study was a well designed, population-based case-control study. Case ascertainment and case survival status were established using the nationwide, high-quality Cancer Registry and Causes of Death Registry in Sweden. Exposure status of the participants was determined using genotyping methods with low error rates from which all results underwent detailed quality control. We sought to obtain tissue samples from the deceased cases and those cases that had declined donation of a blood sample, and were able to obtain the majority of the samples requested. The relative minor lack of tissue accessibility is unlikely to be related to our exposure, *ESR1 *or *EGF *genetic variation, as it depended on the inability of the respective pathology department to retrieve the samples. The tissue sample availability was therefore random and could not have lead to selection bias. The main concern is that the non-participation of a small number of deceased cases might have reduced the power of our study, especially for the survival analysis. Furthermore, a problem might have arisen since we were not able to genotype seven tagSNPs in *ESR1 *and one tagSNP in *EGF *in the tissue samples. If these eight tagSNPs were in fact associated with severe disease, the association with risk of breast cancer death might have been biased towards null in our study since we did not genotype all the severe cases. The fact that the results were not different when we restricted our analyses to the most severe cases among those who donated blood samples indicates that the eight tagSNPs were unlikely to be associated with severe disease.

In the selection stage of our study, we oversampled cases and controls that were long-term users of menopausal hormones and those that had self-reported diabetes mellitus. In the case of an association between the tagSNPs under study and menopausal hormone use or diabetes mellitus, this oversampling might have caused us to detect an artificial association between the tagSNPs and breast cancer risk. We therefore assessed if the tagSNPs were associated with menopausal hormone use or diabetes mellitus. We found no connection between the factors and conclude that the oversampling is unlikely to have posed a problem in our study.

Most previous publications regarding the *ESR1 *gene and breast cancer risk have included only a few polymorphisms in the gene. One study, however, genotyped 17 common SNPs in the *ESR1 *gene and found three haplotypes to decrease breast cancer risk and one haplotype that increased the risk [6]. None of the haplotypes carried SNPs that were located in the region in *ESR1 *we found to be associated with breast cancer risk. Two of the haplotypes that showed a protective effect against breast cancer risk (H4 and H6) carried our TAG21 (rs1801132, codon 325) [6]. We were not able to confirm this association using a window or a haplotype analysis.

The *Pvu*II (TAG6, rs2234693), *Xba*I (TAG7, rs9340799), codon 243 (TAG14, rs4986934) and codon 325 (TAG21, rs1801132) variants are among the most commonly studied polymorphisms in the *ESR1 *gene. The first two have been suggested in a couple of studies to decrease the risk of endometrial cancer [25,26] and *Pvu*II might affect breast cancer survival depending on oestrogen receptor status of the tumour [27], but no consistent effect over studies has been shown for the four variants with regard to breast cancer risk [19,28-37]. We found no association between these SNPs and overall breast cancer risk, NPI or breast cancer survival.

## Conclusion

We analysed common genetic variation in the *ESR1 *and *EGF *genes in relation to breast cancer risk, tumour characteristics and breast cancer survival using a comprehensive haplotype tagging analysis. To our knowledge, this is the first systematic association study of these two genes for breast cancer susceptibility and prognosis. We located a region in *ESR1 *which showed a moderate signal for association with breast cancer risk, but were unable to link common variation in the *EGF *gene with breast cancer aetiology or prognosis.

## Abbreviations

EGF = epidermal growth factor; ESR1 = (o)estrogen receptor 1; HWE = Hardy-Weinberg equilibrium; LD = linkage disequilibrium; MAF = minor allele frequency; NPI = Nottingham Prognostic Index; PLEM = partition ligation expectation maximisation; SNP = single nucleotide polymorphism; tagSNP = tagging single nucleotide polymorphism; TNM = tumour, nodes, metastasis.

## Competing interests

The authors declare that they have no competing interests.

## Authors' contributions

KE, KC, SW, ETL, PH, KH and JL were involved in planning the study. YLL, YQL and CB administrated the genotyping analysis. KE, HD, YL, AS, KH and JL performed the statistical analysis. KE, KH, PH and JL drafted the manuscript, and all the authors approved the manuscript.

## Supplementary Material

Additional file 1 Additional file 1 is a Word file containing tables and figures with genotyping information, linkage disequilibrium maps and association analyses. Click here for file
