# Supplementary material for: ESR1 and EGF genetic variation in relation to breast cancer risk and survival
Source: Breast Cancer Res. 2008 Feb 14;10(1):R15. doi: 10.1186/bcr1861 (PMC2374971; doi:10.1186/bcr1861)
Supplement: Additional file 1 — is a Word file containing tables and figures with genotyping information, linkage disequilibrium maps and association analyses. [file bcr1861-S1.doc]

Supplementary Table 1. Summary data on 228 single nucleotide polymorphisms (SNPs) in *ESR1* and its 20kb flanking sequences that were successfully genotyped in 92 Swedish controls.

| SNP no. | SNP name | Position^a^ | Alleles^b^ | Minor allele  frequency^c^ | HWE  P-value^d^ | tagSNPs |
| --- | --- | --- | --- | --- | --- | --- |
| 1 | rs6914569 | 152161310 | G/C | 0.01 | 0.91 |  |
| 2 | rs12528286 | 152164113 | T | 0 | --- |  |
| 3 | rs6903180 | 152166924 | G/A | 0.53 | 0.38 |  |
| 4 | rs2071454 | 152168517 | T/G | 0.11 | 0.97 |  |
| 5 | rs867239 | 152170324 | C/G | 0.01 | 0.92 |  |
| 6 | rs872921 | 152170632 | G | 0 | --- |  |
| 7 | rs2077647 | 152170770 | A/G | 0.42 | 0.65 |  |
| 8 | rs746432 | 152171001 | C/G | 0.05 | 0.65 |  |
| 9 | rs9479119 | 152171439 | G/T | 0.01 | 0.96 |  |
| 10 | rs532010 | 152172611 | T/C | 0.34 | 0.67 |  |
| 11 | rs7739843 | 152173113 | A | 0 | --- |  |
| 12 | rs576330 | 152173868 | C/T | 0.02 | 0.87 |  |
| 13 | rs10484922 | 152174010 | C/T | 0.09 | 0.73 |  |
| 14 | rs3778609 | 152174880 | C | 0 | --- |  |
| 15 | rs9479120 | 152175760 | A/G | 0.01 | 0.96 |  |
| 16 | rs9340777 | 152177170 | C | 0 | --- |  |
| 17 | rs17081716 | 152179341 | A/G | 0.12 | 0.80 |  |
| 18 | rs6930222 | 152179633 | G/T | 0.01 | 0.96 |  |
| 19 | rs3904766 | 152180531 | G | 0 | --- |  |
| 20 | rs3853248 | 152181579 | T/C | 0.12 | 0.72 | TAG1 |
| 21 | rs3844508 | 152181735 | T | 0 | --- |  |
| 22 | rs9371557 | 152181902 | A/G | 0.06 | 0.53 | TAG2 |
| 23 | rs11753093 | 152186110 | G/A | 0.03 | 0.74 |  |
| 24 | rs6936294 | 152187813 | G | 0 | --- |  |
| 25 | rs3866461 | 152188414 | T/C | 0.12 | 0.81 |  |
| 26 | rs6910339 | 152188911 | G/T | 0.12 | 0.82 |  |
| 27 | rs7775047 | 152193886 | G/C | 0.14 | 0.53 | TAG3 |
| 28 | rs6926750 | 152194681 | A/G | 0.10 | 0.76 |  |
| 29 | rs6903763 | 152194849 | G | 0 | --- |  |
| 30 | rs6909023 | 152195390 | G/A | 0.02 | 0.87 |  |
| 31 | rs827422 | 152198421 | T/C | 0.45 | 0.99 |  |
| 32 | rs827421 | 152198815 | T/C | 0.41 | 0.52 | TAG4 |
| 33 | rs3853250 | 152201593 | A/C | 0.40 | 0.86 | TAG5 |
| 34 | rs4986936 | 152204977 | T | 0 | --- |  |
| 35 | rs2234693 | 152205028 | T/C | 0.40 | 0.82 | TAG6 |
| 36 | rs9340799 | 152205074 | A/G | 0.28 | 0.49 | TAG7 |
| 37 | rs4986937 | 152205204 | G | 0 | --- |  |
| 38 | rs3734807 | 152205698 | A | 0 | --- |  |
| 39 | rs7774230 | 152205932 | C/T | 0.40 | 0.82 |  |
| 40 | rs7739085 | 152206241 | G/C | 0.38 | 1.00 |  |
| 41 | rs9340814 | 152206750 | T | 0 | --- |  |
| 42 | rs9322332 | 152208494 | C/A | 0.41 | 0.95 |  |
| 43 | rs9479130 | 152210149 | A/C | 0.40 | 0.82 |  |
| 44 | rs13437345 | 152212678 | C/T | 0.01 | 0.95 |  |
| 45 | rs4870057 | 152213591 | A/G | 0.29 | 0.03 | TAG8 |
| 46 | rs1709181 | 152216873 | T/C | 0.42 | 0.22 | TAG9 |
| 47 | rs7772626 | 152218173 | C | 0 | --- |  |
| 48 | rs7747344 | 152218597 | G | 0 | --- |  |
| 49 | rs827420 | 152219222 | C/T | 0.37 | 0.37 |  |
| 50 | rs712221 | 152221934 | A/T | 0.39 | 0.29 |  |
| 51 | rs1514348 | 152224008 | A/C | 0.40 | 0.83 |  |
| 52 | rs1709180 | 152225713 | C/T | 0.05 | 0.14 | TAG10 |
| 53 | rs1709183 | 152235689 | A/G | 0.29 | 0.75 | TAG11 |
| 54 | rs1033182 | 152236727 | G/A | 0.35 | 0.97 | TAG12 |
| 55 | rs11155819 | 152241052 | T/C | 0.36 | 0.66 |  |
| 56 | rs6557168 | 152242894 | T/C | 0.41 | 0.19 | TAG13 |
| 57 | rs4986934 | 152243568 | C/T | 0.02 | 0.87 | TAG14 |
| 58 | rs6557170 | 152244797 | G/A | 0.26 | 0.61 | TAG15 |
| 59 | rs6903108 | 152245012 | T | 0 | --- |  |
| 60 | rs4365941 | 152245081 | G/A | 0.04 | 0.66 |  |
| 61 | rs11155820 | 152245903 | A/G | 0.35 | 0.37 | TAG16 |
| 62 | rs1913474 | 152250415 | C/T | 0.25 | 0.78 |  |
| 63 | rs1606678 | 152251508 | G/A | 0.10 | 0.83 |  |
| 64 | rs7761846 | 152254201 | T/C | 0.08 | 0.61 |  |
| 65 | rs12193186 | 152255098 | C/A | 0.01 | 0.91 |  |
| 66 | rs13203472 | 152256637 | C/T | 0.07 | 0.41 |  |
| 67 | rs7773170 | 152256683 | A | 0 | --- |  |
| 68 | rs4870059 | 152265225 | A/G | 0.01 | 0.96 |  |
| 69 | rs12215922 | 152265794 | A/G | 0.34 | 0.11 |  |
| 70 | rs7739316 | 152266616 | G | 0 | --- |  |
| 71 | rs7740131 | 152267077 | G | 0 | --- |  |
| 72 | rs2347923 | 152269114 | A/C | 0.34 | 0.91 |  |
| 73 | rs1514347 | 152271138 | G/A | 0.25 | 0.46 | TAG17 |
| 74 | rs2347867 | 152271543 | A/G | 0.38 | 0.58 |  |
| 75 | rs6557171 | 152276286 | C/T | 0.34 | 0.79 |  |
| 76 | rs4870061 | 152279161 | C/T | 0.26 | 0.64 |  |
| 77 | rs4458702 | 152282116 | G/A | 0.25 | 0.51 |  |
| 78 | rs4305732 | 152282141 | G/A | 0.39 | 0.66 |  |
| 79 | rs988328 | 152282843 | A/G | 0.20 | 0.75 | TAG18 |
| 80 | rs6557172 | 152285997 | C | 0 | --- |  |
| 81 | rs9397456 | 152288845 | G/A | 0.27 | 0.49 |  |
| 82 | rs1606680 | 152290224 | C/G | 0.27 | 0.49 |  |
| 83 | rs12154178 | 152292773 | A/C | 0.41 | 0.71 | TAG19 |
| 84 | rs2347868 | 152293261 | C/T | 0.27 | 0.41 |  |
| 85 | rs12662655 | 152298188 | A/C | 0.01 | 0.91 |  |
| 86 | rs6927072 | 152299380 | G/T | 0.40 | 0.21 |  |
| 87 | rs7739274 | 152300202 | G/A | 0.02 | 0.86 |  |
| 88 | rs6912184 | 152301899 | A/G | 0.27 | 0.68 |  |
| 89 | rs4583998 | 152302361 | G/A | 0.33 | 0.10 | TAG20 |
| 90 | rs6911230 | 152305053 | G/A | 0.31 | 0.29 |  |
| 91 | rs1801132 | 152307215 | C/G | 0.27 | 0.91 | TAG21 |
| 92 | rs6914211 | 152310233 | T/A | 0.11 | 0.91 |  |
| 93 | rs3020314 | 152312365 | T/C | 0.39 | 0.30 | TAG22 |
| 94 | rs3020377 | 152314091 | A/G | 0.42 | 0.14 | TAG23 |
| 95 | rs3020380 | 152315798 | T | 0 | --- |  |
| 96 | rs7745370 | 152315953 | T/C | 0.13 | 0.71 |  |
| 97 | rs3020317 | 152320434 | T/C | 0.23 | 0.69 | TAG24 |
| 98 | rs3003922 | 152321360 | C/G | 0.27 | 0.40 |  |
| 99 | rs7748205 | 152321384 | T/C | 0.13 | 0.67 |  |
| 100 | rs3020395 | 152321497 | A/G | 0.24 | 0.19 |  |
| 101 | rs3020396 | 152321571 | A/G | 0.37 | 0.04 |  |
| 102 | rs7762893 | 152321608 | C | 0 | --- |  |
| 103 | rs3798760 | 152322165 | G | 0 | --- |  |
| 104 | rs1884051 | 152324972 | A/G | 0.38 | 0.03 | TAG25 |
| 105 | rs985191 | 152325151 | A/C | 0.11 | 0.84 |  |
| 106 | rs985192 | 152325171 | C/A | 0.25 | 0.49 |  |
| 107 | rs3003925 | 152326151 | A/G | 0.23 | 0.64 | TAG26 |
| 108 | rs985695 | 152328398 | C/T | 0.17 | 0.62 |  |
| 109 | rs3020318 | 152331463 | C/T | 0.40 | 0.05 | TAG27 |
| 110 | rs1884052 | 152333059 | C/G | 0.18 | 0.51 |  |
| 111 | rs1884053 | 152333159 | T/C | 0.40 | 0.10 |  |
| 112 | rs2982737 | 152333792 | T | 0 | --- |  |
| 113 | rs9383951 | 152337306 | G/C | 0.01 | 0.91 |  |
| 114 | rs7759554 | 152338727 | C/T | 0.01 | 0.92 |  |
| 115 | rs2179922 | 152338793 | G/A | 0.13 | 0.58 |  |
| 116 | rs9397461 | 152339342 | T | 0 | --- |  |
| 117 | rs7760755 | 152341394 | T | 0 | --- |  |
| 118 | rs726281 | 152344271 | A/G | 0.30 | 0.52 | TAG28 |
| 119 | rs7753398 | 152344505 | C/T | 0.05 | 0.62 |  |
| 120 | rs728523 | 152344884 | T/A | 0.01 | 0.91 |  |
| 121 | rs728524 | 152345130 | A/G | 0.05 | 0.62 |  |
| 122 | rs3020407 | 152348954 | A/G | 0.36 | 0.60 | TAG29 |
| 123 | rs2144025 | 152349399 | C/T | 0.22 | 0.94 | TAG30 |
| 124 | rs6912180 | 152352581 | A | 0 | --- |  |
| 125 | rs13192976 | 152354108 | A/T | 0.15 | 0.09 |  |
| 126 | rs9340944 | 152355411 | G/A | 0.14 | 0.28 |  |
| 127 | rs6914107 | 152357150 | G | 0 | --- |  |
| 128 | rs11969635 | 152357356 | G | 0 | --- |  |
| 129 | rs7757956 | 152358833 | T/A | 0.14 | 0.74 |  |
| 130 | rs2223923 | 152359908 | A | 0 | --- |  |
| 131 | rs9340949 | 152360403 | G/A | 0.03 | 0.74 |  |
| 132 | rs9340954 | 152361865 | T/G | 0.31 | 0.24 |  |
| 133 | rs722209 | 152364889 | C/T | 0.11 | 0.02 |  |
| 134 | rs6905370 | 152367890 | G/A | 0.31 | 0.12 | TAG31 |
| 135 | rs13216134 | 152370177 | A/G | 0.15 | 0.06 |  |
| 136 | rs1569788 | 152370309 | T/C | 0.32 | 0.37 |  |
| 137 | rs2207231 | 152371577 | A/G | 0.14 | 0.04 |  |
| 138 | rs11967242 | 152373422 | G | 0 | --- |  |
| 139 | rs13203975 | 152374797 | G/A | 0.15 | 0.11 |  |
| 140 | rs9322349 | 152375556 | T/C | 0.03 | 0.74 |  |
| 141 | rs9340981 | 152375891 | T | 0 | --- |  |
| 142 | rs7755185 | 152381308 | A/G | 0.35 | 0.39 |  |
| 143 | rs2207232 | 152381981 | T/C | 0.13 | 0.02 |  |
| 144 | rs9340994 | 152384406 | A/G | 0.08 | 0.49 | TAG32 |
| 145 | rs3020411 | 152385456 | A/G | 0.37 | 0.36 | TAG33 |
| 146 | rs2982704 | 152389406 | A/T | 0.36 | 0.33 |  |
| 147 | rs2982705 | 152389551 | G/C | 0.37 | 0.63 |  |
| 148 | rs9478265 | 152390594 | G/A | 0.06 | 0.53 |  |
| 149 | rs926778 | 152397475 | C/A | 0.30 | 0.13 | TAG34 |
| 150 | rs926779 | 152397613 | G/A | 0.30 | 0.13 |  |
| 151 | rs3020432 | 152399620 | A/G | 0.40 | 0.77 | TAG35 |
| 152 | rs3020433 | 152400020 | T/G | 0.36 | 0.33 |  |
| 153 | rs9371573 | 152402413 | C/A | 0.37 | 0.36 |  |
| 154 | rs3020364 | 152408811 | A/G | 0.37 | 0.39 |  |
| 155 | rs3020365 | 152409686 | G/T | 0.37 | 0.46 |  |
| 156 | rs2982734 | 152409847 | G/A | 0.37 | 0.33 |  |
| 157 | rs2982735 | 152410380 | C/A | 0.36 | 0.27 |  |
| 158 | rs3020366 | 152410451 | T/C | 0.37 | 0.36 |  |
| 159 | rs9341003 | 152411813 | T | 0 | --- |  |
| 160 | rs6902725 | 152412561 | C/T | 0.14 | 0.04 |  |
| 161 | rs6930355 | 152413084 | T/C | 0.14 | 0.04 |  |
| 162 | rs6557192 | 152414235 | C/A | 0.13 | 0.03 |  |
| 163 | rs6557193 | 152414365 | T/C | 0.11 | 0.00 |  |
| 164 | rs12208351 | 152414504 | G | 0 | --- |  |
| 165 | rs7756369 | 152415604 | G/A | 0.14 | 0.04 |  |
| 166 | rs1884152 | 152418873 | G/A | 0.15 | 0.05 | TAG36 |
| 167 | rs6940499 | 152419876 | T | 0 | --- |  |
| 168 | rs6902385 | 152420325 | T/C | 0.14 | 0.03 |  |
| 169 | rs6941035 | 152420394 | A | 0 | --- |  |
| 170 | rs6941045 | 152420414 | A/C | 0.14 | 0.05 |  |
| 171 | rs9397077 | 152421139 | A/G | 0.13 | 0.02 |  |
| 172 | rs9341015 | 152423418 | C/T | 0.01 | 0.92 |  |
| 173 | rs2273206 | 152424004 | G/T | 0.13 | 0.03 |  |
| 174 | rs974276 | 152424113 | A/G | 0.14 | 0.04 |  |
| 175 | rs974277 | 152424514 | C/T | 0.14 | 0.04 |  |
| 176 | rs3798565 | 152424863 | C/T | 0.14 | 0.04 |  |
| 177 | rs3798568 | 152425076 | G/A | 0.13 | 0.14 |  |
| 178 | rs3778078 | 152425819 | A/C | 0.15 | 0.00 |  |
| 179 | rs3778079 | 152425922 | A/G | 0.14 | 0.01 |  |
| 180 | rs3020372 | 152426621 | T/C | 0.16 | 0.59 | TAG37 |
| 181 | rs3778080 | 152426929 | A/C | 0.13 | 0.15 |  |
| 182 | rs6919225 | 152428583 | T/C | 0.12 | 0.01 |  |
| 183 | rs3778082 | 152429357 | G/A | 0.12 | 0.01 |  |
| 184 | rs3798573 | 152431055 | A/G | 0.14 | 0.04 |  |
| 185 | rs3020375 | 152431661 | A/C | 0.28 | 0.00 |  |
| 186 | rs3020376 | 152431692 | A/G | 0.28 | 0.01 |  |
| 187 | rs3778088 | 152435414 | G/A | 0.14 | 0.04 |  |
| 188 | rs3778089 | 152435454 | G/A | 0.13 | 0.15 |  |
| 189 | rs2747645 | 152438045 | T/C | 0.02 | 0.87 |  |
| 190 | rs12181050 | 152438827 | T/C | 0.13 | 0.03 |  |
| 191 | rs2982896 | 152441186 | C/T | 0.26 | 0.00 |  |
| 192 | rs3822990 | 152447658 | C/T | 0.13 | 0.11 | TAG38 |
| 193 | rs3778090 | 152449721 | C/T | 0.13 | 0.14 |  |
| 194 | rs750686 | 152449819 | G/A | 0.35 | 0.21 |  |
| 195 | rs3778092 | 152449966 | A/G | 0.12 | 0.10 |  |
| 196 | rs3778093 | 152452872 | G/A | 0.12 | 0.08 |  |
| 197 | rs3778094 | 152452891 | C/T | 0.12 | 0.07 |  |
| 198 | rs3778095 | 152452997 | G | 0 | --- |  |
| 199 | rs3020381 | 152453771 | T/A | 0.38 | 0.17 |  |
| 200 | rs2474148 | 152454597 | G/T | 0.33 | 0.50 |  |
| 201 | rs2982900 | 152456685 | C/T | 0.05 | 0.64 | TAG39 |
| 202 | rs3020383 | 152458472 | G/C | 0.06 | 0.23 |  |
| 203 | rs3778099 | 152460268 | T/C | 0.17 | 0.25 | TAG40 |
| 204 | rs2228480 | 152461788 | G/A | 0.16 | 0.02 | TAG41 |
| 205 | rs3020385 | 152462541 | G | 0 | --- |  |
| 206 | rs3798577 | 152462823 | C/T | 0.47 | 0.27 | TAG42 |
| 207 | rs2982901 | 152463013 | C | 0 | --- |  |
| 208 | rs2747648 | 152464028 | T/C | 0.02 | 0.83 |  |
| 209 | rs722029 | 152465114 | G | 0 | --- |  |
| 210 | rs3020388 | 152465279 | G | 0 | --- |  |
| 211 | rs1062577 | 152465598 | T/A | 0.06 | 0.54 | TAG43 |
| 212 | rs2813543 | 152466171 | G/A | 0.25 | 0.42 | TAG44 |
| 213 | rs9341088 | 152467007 | C | 0 | --- |  |
| 214 | rs2747649 | 152468194 | C/G | 0.24 | 0.88 |  |
| 215 | rs1543403 | 152470397 | G/C | 0.49 | 0.83 | TAG45 |
| 216 | rs2813545 | 152474010 | G/C | 0.21 | 0.96 |  |
| 217 | rs910416 | 152474595 | T/C | 0.47 | 0.97 | TAG46 |
| 218 | rs9479226 | 152475708 | C | 0 | --- |  |
| 219 | rs9383963 | 152477851 | T/C | 0.26 | 0.67 |  |
| 220 | rs7450824 | 152479796 | T/C | 0.24 | 0.49 | TAG47 |
| 221 | rs7771845 | 152481871 | G | 0 | --- |  |
| 222 | rs2813552 | 152483609 | A/G | 0.19 | 0.85 | TAG48 |
| 223 | rs12681 | 152485082 | G/A | 0.19 | 0.85 |  |
| 224 | rs2813559 | 152486267 | A/G | 0.21 | 0.97 | TAG49 |
| 225 | rs11757692 | 152490506 | C/A | 0.24 | 0.59 | TAG50 |
| 226 | rs2813565 | 152492547 | T/C | 0.02 | 0.83 |  |
| 227 | rs2295194 | 152495252 | C/G | 0.45 | 0.66 | TAG51 |
| 228 | rs9383609 | 152502067 | T/G | 0.47 | 0.35 | TAG52 |

^a^ dbSNP build 126

^b^ Major alleles given first and minor alleles second

^c^ In 92 controls

^d^ From a χ^2^ test in 92 controls

Supplementary Table 2. Summary data on 104 single nucleotide polymorphisms (SNPs) in *EGF* and its 20kb flanking sequences that were successfully genotyped in 92 Swedish controls.

| SNP no. | SNP name | Position^a^ | Alleles^b^ | Minor allele  frequency^c^ | HWE  P-value^d^ | tagSNPs |
| --- | --- | --- | --- | --- | --- | --- |
| 1 | rs718768 | 111043564 | A/G | 0.30 | 0.05 | TAG1 |
| 2 | rs3756267 | 111050725 | T/C | 0.01 | 0.96 |  |
| 3 | rs3756266 | 111050858 | G | 0 | --- |  |
| 4 | rs3756264 | 111051085 | G | 0 | --- |  |
| 5 | rs11568835 | 111052119 | G/A | 0.01 | 0.96 |  |
| 6 | rs11098052 | 111052454 | C | 0 | --- |  |
| 7 | rs4444903 | 111053559 | A/G | 0.41 | 0.21 |  |
| 8 | rs11568849 | 111053986 | A | 0 | --- |  |
| 9 | rs881878 | 111055497 | G/A | 0.37 | 0.06 | TAG2 |
| 10 | rs2250724 | 111056736 | C/T | 0.03 | 0.75 |  |
| 11 | rs2282782 | 111056831 | A | 0 | --- |  |
| 12 | rs4141078 | 111060316 | G/A | 0.04 | 0.70 |  |
| 13 | rs2237043 | 111061858 | T | 0 | --- |  |
| 14 | rs2024114 | 111062333 | A/C | 0.32 | 0.04 |  |
| 15 | rs2237044 | 111062877 | C/G | 0.01 | 0.96 |  |
| 16 | rs12503113 | 111065156 | T/C | 0.40 | 0.06 |  |
| 17 | rs2298982 | 111066245 | A/G | 0.04 | 0.70 |  |
| 18 | rs11568865 | 111069925 | G | 0 | --- |  |
| 19 | rs3796949 | 111073765 | G | 0 | --- |  |
| 20 | rs3822288 | 111073993 | G/A | 0.40 | 0.19 | TAG3 |
| 21 | rs11568877 | 111080310 | G | 0 | --- |  |
| 22 | rs3822287 | 111081092 | C/T | 0.01 | 0.96 |  |
| 23 | rs11568895 | 111083502 | T/C | 0.03 | 0.74 |  |
| 24 | rs7655579 | 111085157 | A/G | 0.39 | 0.20 |  |
| 25 | rs4698756 | 111085891 | G/A | 0.38 | 0.10 |  |
| 26 | rs2282784 | 111088695 | G/A | 0.38 | 0.16 | TAG4 |
| 27 | rs3796946 | 111090960 | A/G | 0.37 | 0.21 |  |
| 28 | rs2298983 | 111093822 | T/C | 0.41 | 0.16 |  |
| 29 | rs1024599 | 111094562 | C | 0 | --- |  |
| 30 | rs1024600 | 111095039 | C/G | 0.33 | 0.17 | TAG5 |
| 31 | rs2298985 | 111096018 | A | 0 | --- |  |
| 32 | rs2067004 | 111099813 | T/C | 0.38 | 0.07 |  |
| 33 | rs3764861 | 111104691 | A/T | 0.38 | 0.10 |  |
| 34 | rs2190907 | 111105317 | T/C | 0.39 | 0.44 |  |
| 35 | rs2298986 | 111105598 | C/T | 0.03 | 0.79 |  |
| 36 | rs10023272 | 111106253 | T/C | 0.38 | 0.31 |  |
| 37 | rs11568970 | 111108304 | C | 0 | --- |  |
| 38 | rs4698802 | 111109384 | T/A | 0.01 | 0.96 |  |
| 39 | rs2298988 | 111110349 | T | 0 | --- |  |
| 40 | rs2255355 | 111110992 | A/C | 0.01 | 0.91 |  |
| 41 | rs2298989 | 111111122 | T/C | 0.41 | 0.27 |  |
| 42 | rs7670908 | 111112074 | G/A | 0.04 | 0.66 | TAG6 |
| 43 | rs2237046 | 111113068 | T | 0 | --- |  |
| 44 | rs2237047 | 111113426 | A | 0 | --- |  |
| 45 | rs9991367 | 111113750 | C/T | 0.04 | 0.66 | TAG7 |
| 46 | rs2237048 | 111113851 | C | 0 | --- |  |
| 47 | rs2298993 | 111114562 | G/A | 0.42 | 0.29 |  |
| 48 | rs6811428 | 111114882 | A | 0 | --- |  |
| 49 | rs9995637 | 111116336 | T/G | 0.05 | 0.66 |  |
| 50 | rs11568995 | 111117034 | G/A | 0.04 | 0.70 |  |
| 51 | rs2237049 | 111120240 | G/A | 0.39 | 0.89 |  |
| 52 | rs2237050 | 111120267 | A | 0 | --- |  |
| 53 | rs2302135 | 111120596 | A | 0 | --- |  |
| 54 | rs2237051 | 111120647 | G/A | 0.39 | 0.62 |  |
| 55 | rs6533484 | 111121102 | T | 0 | --- |  |
| 56 | rs7695064 | 111121306 | A | 0 | --- |  |
| 57 | rs6824594 | 111123897 | C/T | 0.39 | 0.86 |  |
| 58 | rs2298995 | 111125425 | A | 0 | --- |  |
| 59 | rs2298996 | 111125906 | G/A | 0.04 | 0.70 |  |
| 60 | rs2298998 | 111126605 | T | 0 | --- |  |
| 61 | rs2074390 | 111129465 | C/T | 0.39 | 0.61 |  |
| 62 | rs2237053 | 111130529 | T/C | 0.39 | 0.61 |  |
| 63 | rs2237054 | 111130638 | T/A | 0.04 | 0.70 |  |
| 64 | rs6827914 | 111130758 | C/T | 0.04 | 0.00 |  |
| 65 | rs7692976 | 111131016 | A/G | 0.44 | 0.29 | TAG8 |
| 66 | rs6815092 | 111132325 | A | 0 | --- |  |
| 67 | rs4698803 | 111133876 | T/A | 0.21 | 0.53 | TAG9 |
| 68 | rs11569087 | 111135629 | G | 0 | --- |  |
| 69 | rs11569088 | 111135684 | C | 0 | --- |  |
| 70 | rs882481 | 111135887 | T | 0 | --- |  |
| 71 | rs2299001 | 111136936 | T/C | 0.03 | 0.78 |  |
| 72 | rs2299002 | 111137075 | A | 0 | --- |  |
| 73 | rs2074389 | 111140173 | C | 0 | --- |  |
| 74 | rs11569098 | 111140405 | C | 0 | --- |  |
| 75 | rs4698757 | 111141643 | T | 0 | --- |  |
| 76 | rs2282786 | 111141945 | T | 0 | --- |  |
| 77 | rs12507356 | 111142777 | G | 0 | --- |  |
| 78 | rs11569105 | 111144158 | G | 0 | --- |  |
| 79 | rs3733626 | 111144941 | G/T | 0.04 | 0.70 |  |
| 80 | rs971696 | 111147314 | T/A | 0.04 | 0.70 |  |
| 81 | rs971695 | 111147377 | C/T | 0.04 | 0.70 |  |
| 82 | rs2074391 | 111149178 | G/A | 0.01 | 0.96 |  |
| 83 | rs3822285 | 111149575 | C | 0 | --- |  |
| 84 | rs3796941 | 111149662 | T/C | 0.01 | 0.96 |  |
| 85 | rs2282787 | 111150243 | A/G | 0.05 | 0.58 |  |
| 86 | rs7653900 | 111153304 | T/C | 0.07 | 0.30 | TAG10 |
| 87 | rs10021697 | 111154472 | G/T | 0.06 | 0.57 | TAG11 |
| 88 | rs11569161 | 111154666 | A | 0 | --- |  |
| 89 | rs17041230 | 111156964 | C/T | 0.06 | 0.53 |  |
| 90 | rs9993323 | 111160155 | G | 0 | --- |  |
| 91 | rs7699288 | 111161726 | G/C | 0.35 | 0.50 |  |
| 92 | rs6533487 | 111165268 | C/A | 0.35 | 0.17 |  |
| 93 | rs7440841 | 111165698 | C/A | 0.35 | 0.50 |  |
| 94 | rs10488883 | 111167331 | A/G | 0.01 | 0.96 |  |
| 95 | rs17320827 | 111167460 | G/T | 0.43 | 0.66 | TAG12 |
| 96 | rs730598 | 111171076 | A/G | 0.05 | 0.61 | TAG13 |
| 97 | rs4698805 | 111174942 | C/G | 0.40 | 0.23 |  |
| 98 | rs6816508 | 111176792 | T/A | 0.40 | 0.23 |  |
| 99 | rs6533489 | 111180514 | A/G | 0.41 | 0.18 | TAG14 |
| 100 | rs10012953 | 111183811 | A/G | 0.32 | 0.95 |  |
| 101 | rs10454793 | 111185612 | G/A | 0.04 | 0.68 | TAG15 |
| 102 | rs12108641 | 111188048 | G/T | 0.14 | 0.80 |  |
| 103 | rs6837303 | 111189034 | C/T | 0.38 | 0.91 |  |
| 104 | rs17041272 | 111189531 | C | 0 | --- |  |

^a^ dbSNP build 126

^b^ Major alleles given first and minor alleles second

^c^ In 92 controls

^d^ From a χ^2^ test in 92 controls

Supplementary Table 3. Summary of the tagSNPs in *ESR1* and *EGF* that were genotyped in all breast cancer cases and controls.

| tagSNPs | SNP name | Number of  cases/controls | MAF^a^ | HWE  *P*-value^a,b^ |  | tagSNPs | SNP name | Number of cases/controls | MAF^a^ | HWE  *P*-value^a,b^ |
| --- | --- | --- | --- | --- | --- | --- | --- | --- | --- | --- |
| **ESR1** |  |  |  |  |  | TAG35 | rs3020432 | 1541/1500 | 0.38 | 0.88 |
| TAG1 | rs3853248 | 1488/1463 | 0.14 | 0.79 |  | TAG36 | rs1884152 | 1534/1484 | 0.14 | 0.91 |
| TAG2 | rs9371557 | 1491/1436 | 0.03 | 0.19 |  | TAG37 | rs3020372 | 1541/1499 | 0.18 | 0.53 |
| TAG3 | rs7775047 | 1271/1474 | 0.15 | 0.90 |  | TAG38 | rs3822990 | 1560/1498 | 0.12 | 0.22 |
| TAG4 | rs827421 | 1535/1486 | 0.49 | 0.10 |  | TAG39 | rs2982900 | 1561/1494 | 0.05 | 0.83 |
| TAG5 | rs3853250 | 1541/1493 | 0.47 | 0.15 |  | TAG40 | rs3778099 | 1485/1460 | 0.14 | 0.07 |
| TAG6^c^ | rs2234693 | 1557/1511 | 0.47 | 0.30 |  | TAG41 | rs2228480 | 1547/1486 | 0.19 | 0.75 |
| TAG7^d^ | rs9340799 | 1555/1512 | 0.34 | 0.90 |  | TAG42 | rs3798577 | 1552/1478 | 0.49 | 0.93 |
| TAG8 | rs4870057 | 1542/1487 | 0.34 | 0.63 |  | TAG43 | rs1062577 | 1533/1477 | 0.06 | 0.84 |
| TAG9 | rs1709181 | 1528/1475 | 0.42 | 0.40 |  | TAG44 | rs2813543 | 1138/1338 | 0.24 | 0.48 |
| TAG10 | rs1709180 | 1551/1482 | 0.07 | 0.85 |  | TAG45 | rs1543403 | 1515/1480 | 0.50 | 0.57 |
| TAG11 | rs1709183 | 1548/1496 | 0.28 | 1.00 |  | TAG46 | rs910416 | 1545/1498 | 0.44 | 0.44 |
| TAG12 | rs1033182 | 1508/1463 | 0.37 | 0.95 |  | TAG47 | rs7450824 | 1534/1477 | 0.27 | 0.31 |
| TAG13 | rs6557168 | 1266/1472 | 0.35 | 0.67 |  | TAG48 | rs2813552 | 1515/1449 | 0.21 | 0.29 |
| TAG14^e^ | rs4986934 | 1561/1513 | 0.04 | 0.20 |  | TAG49 | rs2813559 | 1510/1481 | 0.20 | 0.11 |
| TAG15 | rs6557170 | 1548/1491 | 0.23 | 0.24 |  | TAG50 | rs11757692 | 1544/1492 | 0.23 | 0.24 |
| TAG16 | rs11155820 | 1548/1488 | 0.32 | 0.73 |  | TAG51 | rs2295194 | 1547/1490 | 0.44 | 0.55 |
| TAG17 | rs1514347 | 1148/1346 | 0.25 | 0.04 |  | TAG52 | rs9383609 | 1497/1475 | 0.40 | 0.53 |
| TAG18 | rs988328 | 1543/1493 | 0.15 | 0.81 |  |  |  |  |  |  |
| TAG19 | rs12154178 | 1519/1477 | 0.28 | 0.56 |  | **EGF** |  |  |  |  |
| TAG20 | rs4583998 | 1147/1339 | 0.30 | 0.02 |  | TAG1 | rs718768 | 1555/1494 | 0.26 | 0.01 |
| TAG21^f^ | rs1801132 | 1561/1513 | 0.23 | 0.86 |  | TAG2 | rs881878 | 1540/1497 | 0.32 | 0.50 |
| TAG22 | rs3020314 | 1298/1502 | 0.33 | 0.74 |  | TAG3 | rs3822288 | 1547/1498 | 0.32 | 0.29 |
| TAG23 | rs3020377 | 1546/1505 | 0.33 | 0.55 |  | TAG4 | rs2282784 | 1557/1485 | 0.39 | 0.15 |
| TAG24 | rs3020317 | 1514/1469 | 0.21 | 0.72 |  | TAG5 | rs1024600 | 1502/1440 | 0.29 | 0.27 |
| TAG25 | rs1884051 | 1545/1481 | 0.32 | 0.39 |  | TAG6 | rs7670908 | 1553/1489 | 0.07 | 0.45 |
| TAG26 | rs3003925 | 1527/1458 | 0.19 | 0.40 |  | TAG7 | rs9991367 | 1476/1464 | 0.06 | 0.79 |
| TAG27 | rs3020318 | 1545/1498 | 0.34 | 0.62 |  | TAG8 | rs7692976 | 1485/1451 | 0.41 | 0.89 |
| TAG28 | rs726281 | 1468/1422 | 0.26 | 0.77 |  | TAG9 | rs4698803 | 1533/1482 | 0.25 | 0.07 |
| TAG29 | rs3020407 | 1551/1495 | 0.32 | 0.50 |  | TAG10 | rs7653900 | 1255/1438 | 0.08 | 0.32 |
| TAG30 | rs2144025 | 1116/1311 | 0.18 | 0.04 |  | TAG11 | rs10021697 | 1468/1436 | 0.05 | 0.31 |
| TAG31 | rs6905370 | 1543/1490 | 0.31 | 0.75 |  | TAG12 | rs17320827 | 1527/1486 | 0.34 | 0.29 |
| TAG32 | rs9340994 | 1552/1500 | 0.05 | 0.27 |  | TAG13 | rs730598 | 1556/1496 | 0.07 | 0.40 |
| TAG33 | rs3020411 | 1519/1488 | 0.38 | 0.54 |  | TAG14 | rs6533489 | 1501/1461 | 0.35 | 0.19 |
| TAG34 | rs926778 | 1511/1452 | 0.32 | 0.86 |  | TAG15 | rs10454793 | 1514/1465 | 0.04 | 0.32 |

^a^ Among all controls

^b^ Chi-square tests

^c^ Also named pvuII

^d^ Also named xbaI

^e^ Also named codon 243

^f^ Also named codon 325

MAF = Minor allele frequency, HWE = Hardy-Weinberg Equilibrium

Supplementary Table 4. Summary of the association results (*P*-values) between tagSNPs and breast cancer risk, NPI and breast cancer survival from the single-SNP and 5-SNP genotype analyses.

| SNP |  | Risk | |  | NPI | |  | Survival | |
| --- | --- | --- | --- | --- | --- | --- | --- | --- | --- |
|  |  | Single-SNP | 5-SNP |  | Single-SNP | 5-SNP |  | Single-SNP | 5-SNP |
| TAG1 |  | 0.457 |  |  | 0.05 |  |  | 0.814 |  |
| TAG2 |  | 0.043 |  |  | 0.654 |  |  | 0.717 |  |
| TAG3 |  | 0.191 | 0.074 |  | 0.01 | 0.274 |  | 0.499 | 0.788 |
| TAG4 |  | 0.366 | 0.057 |  | 0.787 | 0.239 |  | 0.384 | 0.795 |
| TAG5 |  | 0.04 | 0.405 |  | 0.949 | 0.236 |  | 0.688 | 0.833 |
| TAG6 |  | 0.136 | 0.162 |  | 0.822 | 0.41 |  | 0.851 | 0.318 |
| TAG7 |  | 0.076 | 0.21 |  | 0.096 | 0.32 |  | 0.865 | 0.716 |
| TAG8 |  | 0.041 | 0.452 |  | 0.143 | 0.236 |  | 0.93 | 0.934 |
| TAG9 |  | 0.083 | 0.239 |  | 0.378 | 0.687 |  | 0.634 | 0.958 |
| TAG10 |  | 0.719 | 0.167 |  | 0.999 | 0.354 |  | 0.379 | 0.631 |
| TAG11 |  | 0.546 | 0.387 |  | 0.79 | 0.035 |  | 0.418 | 0.38 |
| TAG12 |  | 0.207 | 0.433 |  | 0.18 | 0.051 |  | 0.238 | 0.356 |
| TAG13 |  | 0.156 | 0.266 |  | 0.044 | 0.087 |  | 0.464 | 0.628 |
| TAG14 |  | 0.396 | 0.274 |  | 0.629 | 0.074 |  | 0.568 | 0.302 |
| TAG15 |  | 0.067 | 0.305 |  | 0.354 | 0.177 |  | 0.827 | 0.621 |
| TAG16 |  | 0.896 | 0.25 |  | 0.199 | 0.575 |  | 0.074 | 0.513 |
| TAG17 |  | 0.056 | 0.128 |  | 0.779 | 0.39 |  | 0.751 | 0.608 |
| TAG18 |  | 0.404 | 0.102 |  | 0.38 | 0.585 |  | 0.647 | 0.575 |
| TAG19 |  | 0.9 | 0.027 |  | 0.591 | 0.842 |  | 0.699 | 0.445 |
| TAG20 |  | 0.925 | 0.089 |  | 0.488 | 0.645 |  | 0.24 | 0.399 |
| TAG21 |  | 0.057 | 0.119 |  | 0.375 | 0.418 |  | 0.054 | 0.342 |
| TAG22 |  | 0.172 | 0.103 |  | 0.258 | 0.307 |  | 0.925 | 0.393 |
| TAG23 |  | 0.074 | 0.369 |  | 0.279 | 0.099 |  | 0.988 | 0.903 |
| TAG24 |  | 0.03 | 0.034 |  | 0.919 | 0.179 |  | 0.613 | 0.806 |
| TAG25 |  | 0.308 | 0.077 |  | 0.668 | 0.059 |  | 0.731 | 0.391 |
| TAG26 |  | 0.945 | 0.056 |  | 0.668 | 0.365 |  | 0.702 | 0.921 |
| TAG27 |  | 0.422 | 0.18 |  | 0.843 | 0.559 |  | 0.317 | 0.927 |
| TAG28 |  | 0.49 | 0.001 |  | 0.762 | 0.978 |  | 0.191 | 0.606 |
| TAG29 |  | 0.692 | 0.004 |  | 0.869 | 0.986 |  | 0.327 | 0.432 |
| TAG30 |  | 0.198 | 0.024 |  | 0.872 | 0.775 |  | 0.369 | 0.458 |
| TAG31 |  | 0.715 | 0.045 |  | 0.62 | 0.651 |  | 0.03 | 0.407 |
| TAG32 |  | 0.081 | 0.297 |  | 0.132 | 0.717 |  | 0.124 | 0.403 |
| TAG33 |  | 0.21 | 0.112 |  | 0.211 | 0.281 |  | 0.046 | 0.319 |
| TAG34 |  | 0.605 | 0.083 |  | 0.221 | 0.185 |  | 0.237 | 0.258 |
| TAG35 |  | 0.212 | 0.078 |  | 0.171 | 0.25 |  | 0.022 | 0.036 |
| TAG36 |  | 0.423 | 0.162 |  | 0.876 | 0.548 |  | 0.066 | 0.008 |
| TAG37 |  | 0.628 | 0.104 |  | 0.067 | 0.464 |  | 0.992 | 0.064 |
| TAG38 |  | 0.392 | 0.605 |  | 0.653 | 0.333 |  | 0.36 | 0.076 |
| TAG39 |  | 0.182 | 0.713 |  | 0.193 | 0.29 |  | 0.706 | 0.635 |
| TAG40 |  | 0.317 | 0.62 |  | 0.286 | 0.513 |  | 0.945 | 0.447 |
| TAG41 |  | 0.67 | 0.623 |  | 0.719 | 0.702 |  | 0.791 | 0.322 |
| TAG42 |  | 0.778 | 0.825 |  | 0.977 | 0.754 |  | 0.389 | 0.363 |
| TAG43 |  | 0.474 | 0.979 |  | 0.96 | 0.801 |  | 0.033 | 0.482 |
| TAG44 |  | 0.439 | 0.646 |  | 0.814 | 0.957 |  | 0.04 | 0.232 |
| TAG45 |  | 0.701 | 0.39 |  | 0.462 | 0.949 |  | 0.308 | 0.218 |
| TAG46 |  | 0.968 | 0.114 |  | 0.984 | 0.415 |  | 0.284 | 0.099 |
| TAG47 |  | 0.385 | 0.188 |  | 0.904 | 0.159 |  | 0.654 | 0.208 |
| TAG48 |  | 0.411 | 0.251 |  | 0.224 | 0.069 |  | 0.707 | 0.429 |
| TAG49 |  | 0.564 | 0.142 |  | 0.251 | 0.123 |  | 0.797 | 0.368 |
| TAG50 |  | 0.996 | 0.332 |  | 0.173 | 0.141 |  | 0.703 | 0.025 |
| TAG51 |  | 0.228 |  |  | 0.412 |  |  | 0.109 |  |
| TAG52 |  | 0.661 |  |  | 0.27 |  |  | 0.511 |  |

*P*-values are obtained from Likelihood ratio tests based on using single tagSNPs (columns 1,3,5) and sliding windows of 5 tagSNPs (columns 2,4,6) as main effects in logistic regression models.


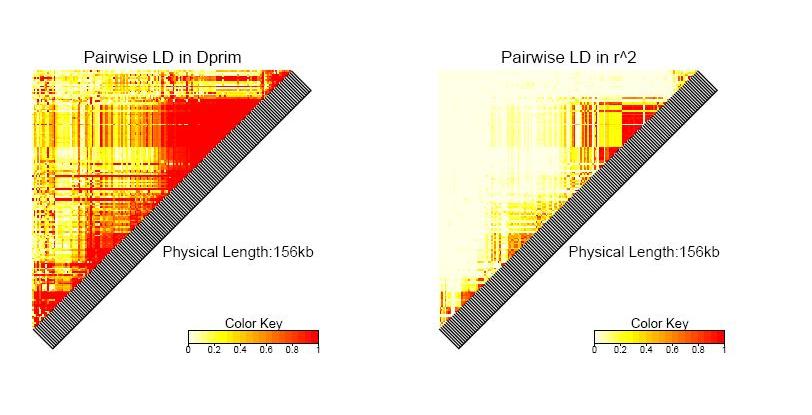


Supplementary Figure 1. LD plots (*D´* and *R^2^*) of 157 SNPs in *ESR1* genotyped in 92 controls and included in our study.


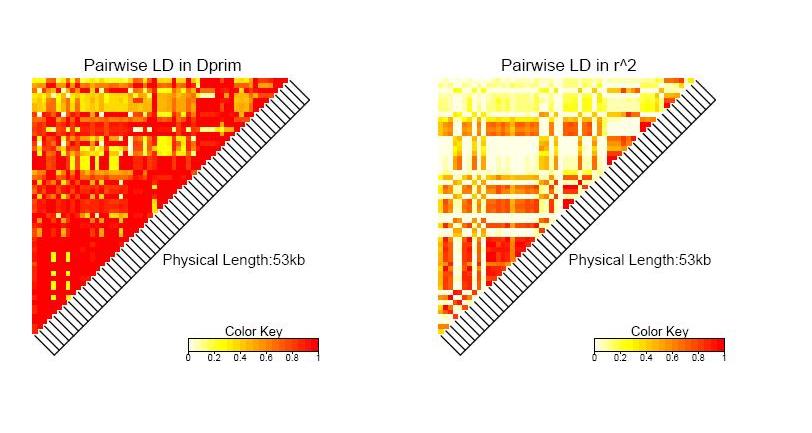


Supplementary Figure 2. LD plots (*D´* and *R^2^*) of 54 SNPs in *EGF* genotyped in 92 controls and included in our study.


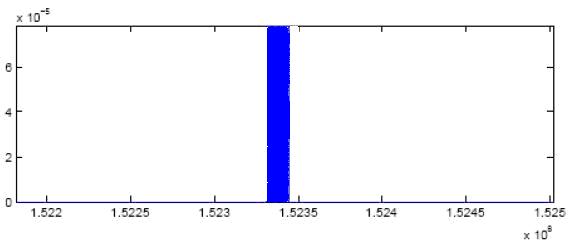


Supplementary Figure 3. Posterior distribution for position of a possible disease mutation.

X-axis: Chromosomal position. Y-axis: Posterior probability density for a disease locus.


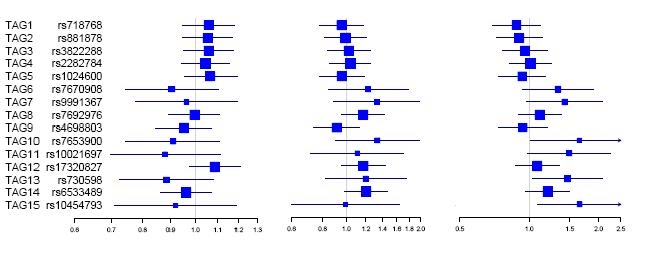


Supplementary Figure 4. Association of the 15 tagSNPs in *EGF* with breast cancer risk (left column), NPI (case only analysis, middle column) and breast cancer survival (right column).

Squares and horizontal lines represent odds and hazard (survival analysis) ratios (change in risk with each addition of the rare allele) and their confidence intervals. Sizes of the squares reflect the minor allele frequencies. NPI was categorized into ≤4 or >4. NPI = Nottingham Prognostic Index.
